# Determination of Heavy Metal Levels and Assessment of *L. monocytogenes* and *Salmonella* spp. Presence in Fishery Products and Mussels from the Marmara Region, Türkiye

**DOI:** 10.3390/toxics13030153

**Published:** 2025-02-23

**Authors:** Esra Akkaya, Karlo Muratoglu, Duygu Tarhan, Nural Pastaci Ozsobaci, Alev Meltem Ercan, Hilal Colak, Hamparsun Hampikyan, Enver Baris Bingol, Mehmet Erman Or, Egon Andoni, Enkeleda Ozuni, Marco Gobbi, Linda Petrucci, Federica Di Cesare, Petra Cagnardi, Giulio Curone, Claudia Maria Balzaretti, Valerio Giaccone, Marta Castrica

**Affiliations:** 1 Department of Food Hygiene and Technology, Faculty of Veterinary Medicine, İstanbul University-Cerrahpaşa, İstanbul 34320, Türkiye; esra.akkaya@iuc.edu.tr (E.A.); hcolak@iuc.edu.tr (H.C.); bingolb@iuc.edu.tr (E.B.B.); 2 Department of Biophysics, School of Medicine, Bahcesehir University, Göztepe, Istanbul 34734, Türkiye; duygu.tarhan@bau.edu.tr; 3 Department of Biophysics, Cerrahpaşa Faculty of Medicine, İstanbul University-Cerrahpaşa, İstanbul 34098, Türkiye; n.pastaciozsobaci@iuc.edu.tr (N.P.O.); meltem@iuc.edu.tr (A.M.E.); 4 Department of Gastronomy and Culinary Arts, Faculty of Fine Arts, Istanbul Beykent University, Istanbul 34500, Türkiye; hamparsun@beykent.edu.tr; 5 Department of Internal Medicine, Faculty of Veterinary Medicine, İstanbul University-Cerrahpaşa, Istanbul 34320, Türkiye; ermanor@iuc.edu.tr; 6 Faculty of Veterinary Medicine, Agricultural University of Tirana, Koder Kamez, 1029 Tirana, Albania; eandoni@ubt.edu.al (E.A.); enkeleda.ozuni@ubt.edu.al (E.O.); 7 Istituto Zooprofilattico Sperimentale dell’Umbria e delle Marche “Togo Rosati”, Via Salvemini 1, 06126 Perugia, Italy; m.gobbi@izsum.it (M.G.); l.petrucci@izsum.it (L.P.); 8 Department of Veterinary Medicine and Animal Sciences, University of Milan, Via dell’Università 6, 26900 Lodi, Italy; federica.dicesare@unimi.it (F.D.C.); petra.cagnardi@unimi.it (P.C.); giulio.curone@unimi.it (G.C.); claudia.balzaretti@unimi.it (C.M.B.); 9 Department of Animal Medicine, Production and Health, University of Padova, Agripolis, Viale dell’Università 16, 35020 Legnaro, Italy; valerio.giaccone@unipd.it; 10 Department of Comparative Biomedicine and Food Science, University of Padova, Agripolis, Viale dell’Università 16, 35020 Legnaro, Italy

**Keywords:** heavy metal, HI, ICP-OES, pathogenic microorganism, risk ranger, THQ

## Abstract

This study evaluated heavy metal levels (Pb, Cd, Hg, As, Cu) and the presence of *Salmonella* spp. and *Listeria monocytogenes* in mussels and commonly consumed fishery products from the Marmara region of Türkiye. Health risks were evaluated using total hazard quotient (THQ) and hazard index (HI) values, while microbial risks in fresh and ready-to-eat (RTE) products were estimated via the Risk Ranger tool. Among 625 samples, Hg (36.96%; CI95 = 33.27–48.81), Pb (9.76%; CI95 = 7.67–12.34), and Cd (19.36%; CI95 = 16.45–22.64) exceeded permissible limits, except in crabs, which remained compliant. Anchovy, sardines, bluefish, shrimps, and octopus met EU Cd limits. Shrimps exhibited higher As_in_ levels than crabs (*p* < 0.05), while squids had significantly higher As_in_ than octopus but lower Pb (*p* < 0.05). Microbiological analysis detected *Salmonella* spp. in 4.00% of samples (CI95 = 2.50–6.30) and *L. monocytogenes* in 4.24% (CI95 = 2.70–6.59). Surmullet, bluefish, red mullet, crabs, mussels, and octopus tested negative for both, while anchovy was negative for *Salmonella* spp. only. THQ and HI assessments emphasized the need for environmental monitoring to mitigate heavy metal contamination. The detection of pathogens highlights the importance of stringent surveillance measures to ensure the safety of fishery products and bivalves.

## 1. Introduction

Fishery products and bivalves are recognized as a vital part of the human diet around the world, being rich sources of proteins, omega-3 fatty acids, vitamins, and minerals [1]. Fishery products, and particularly bivalve mollusks, are of critical importance for public health due to their filter-feeding nature [2]. Therefore, the consumption of fishery products may carry some health risks because these products can become contaminated with environmental pollutants and pathogenic microorganism [3,4,5]. Heavy metals (HMs) are significant environmental pollutants, often introduced into aquatic ecosystems through industrial discharge, agricultural runoff, or atmospheric deposition [6]. Once in the water, these metals can bioaccumulate in the tissues of marine organisms [4,7]. Consumption of fishery products and mussels contaminated with HMs such as mercury (Hg), lead (Pb), cadmium (Cd), and arsenic (As), can result in metal accumulation in human tissues and organs, potentially leading to severe health issues [5,8]. Chronic exposure to heavy metals has been linked to various adverse health effects, including neurological disorders (e.g., mercury poisoning), kidney damage (e.g., cadmium), and cancer (e.g., arsenic) [9]. The International Agency for Research on Cancer (IARC) classifies As and Cd as Group 1 carcinogens, lead as Group 2A, and mercury as Group 2B [10]. Hg is released into the environment primarily through industrial activities, which is subsequently deposited in aquatic ecosystems via atmospheric precipitation [11]. Once in water, Hg is converted by microorganisms in the sediment to methylmercury, a highly toxic organic form that accumulates in marine organisms, particularly in long-living fish such as tuna and king mackerel [12,13,14]. Lead contamination in aquatic environments results from industrial processes such as mining and the historical use of leaded petrol. This metal accumulates in sediments and is absorbed by marine organisms such as oysters, clams, and mussels, which are particularly prone to lead accumulation due to their filter-feeding behavior [15,16]. Cadmium is released into the environment by industrial processes such as metal smelting and phosphate fertilizer production. It enters aquatic systems through agricultural runoff and is absorbed by aquatic organisms [17,18]. Fish can accumulate Cd, while bivalve mollusks exhibit high cadmium concentrations due to their filtration mechanisms [19]. Arsenic, found in the crust of the Earth, can enter water bodies through natural processes such as volcanic activity, rock weathering, and agricultural activities. Additionally, industrial discharge (e.g., wood preservation) contributes to arsenic contamination in aquatic environments [6]. While organic arsenic species are predominantly found in crustaceans such as shrimps and crabs, inorganic arsenic, which is more toxic, has been detected in fish species such as cod and haddock [6,20]. To mitigate health risks, governments and international health agencies have established maximum levels (MLs) for certain contaminants in food for HMs and conduct monitoring programs. The European Commission (EC) has set MLs for Pb at 0.3 mg/kg in fish and 1.5 mg/kg in bivalve mollusks, for Cd at 0.05 mg/kg in fish and 1 mg/kg in bivalve mollusks, and for Hg at 0.5–1 mg/kg in fish species and bivalve mollusks, as stipulated in Regulation (EU) No. 915/2023 (consolidated text). In addition to chemical contaminants, fishery products and bivalve mollusks can serve as a vehicle for various pathogenic microorganisms. Some pathogens are naturally present in marine environments, while others, including those resistant to antimicrobials, can enter aquatic ecosystems through various pathways associated with human and animal activities. These sources include human settlements, agricultural and livestock systems, slaughterhouses, and processing industries. Once introduced into water bodies, these microorganisms have the potential to contaminate fishery products and bivalve mollusks. Furthermore, contamination may persist throughout subsequent stages such as harvesting, handling, packaging, processing, and distribution, particularly when inadequate hygiene and sanitation practices are employed beyond the farm level [21,22]. Bacterial, viral, and parasitic contaminants in fishery products and bivalves can cause foodborne illnesses, which pose a significant threat, particularly to vulnerable populations such as pregnant women, the elderly, and immunocompromised individuals [23,24,25]. According to Regulation (EU) No. 2073/2005 (consolidated text), the absence of *Salmonella* spp. is established as a food safety criterion for cooked crustaceans, molluscan shellfish, and live bivalve mollusks. Concerning *Listeria monocytogenes* (*Lm*) in ready-to-eat (RTE) products, the regulation distinguishes between two scenarios: (i) for RTE foods that support the growth of *Lm*, its absence in 25 g of the product is required; (ii) for RTE foods where growth is not supported, a maximum limit of 100 CFU/g is permissible. However, for RTE products capable of supporting *Lm* growth, a quantitative limit of 100 CFU/g may be applied if the producer can demonstrate to the satisfaction of the competent authorities that this limit will not be exceeded throughout the product’s shelf-life.

The Marmara Sea of Türkiye is one of the most important areas for aquaculture production, utilizing rich fishery resources for commercial (export) and local markets. Marine life in this region is determined by factors such as water temperature and water salinity, as well as pollution levels, all of which contribute to ensuring the diversity of fishery products and bivalve mollusks in the markets. This valuable ecosystem may be threatened by certain pollutants due to various anthropogenic impacts such as high population growth, industrial developments, and agricultural activities [26,27]. The sediments in the Sea of Marmara—terrigenous, biogenic, and authigenic—play a crucial role in water quality, ecosystem health, and nutrient cycling. These sediments originate from river transport, biological contributions, and organic-rich muds, shaping the marine environment. Due to its size and depth, the Marmara Sea has an anoxic bottom water that fosters a eutrophic environment that makes it highly vulnerable to pollution [28,29,30,31,32,33]. Considering these pollution factors, the monitoring of fishery products and bivalve mollusks in the Marmara Sea in terms of microorganisms and heavy metal concentrations is of crucial importance. 

Therefore, this study aimed to determine the levels of heavy metals (Pb, Cd, Hg, As, and Cu) and the presence of *Salmonella* spp. and *Listeria monocytogenes* in mussels and in commonly consumed fishery products, including various fish species, crustaceans, and cephalopods, collected from the Marmara region of Türkiye. Additionally, the potential health risks associated with the consumption of these products were assessed by estimating the total hazard quotient (THQ) and hazard index (HI) values concerning the detected heavy metal levels. Furthermore, the risk posed by *Salmonella* spp. and *L. monocytogenes* in fresh and ready-to-eat (RTE) fishery products was evaluated using the Risk Ranger tool.

## 2. Materials and Methods

### 2.1. Sample Collection

**Figure 1:**
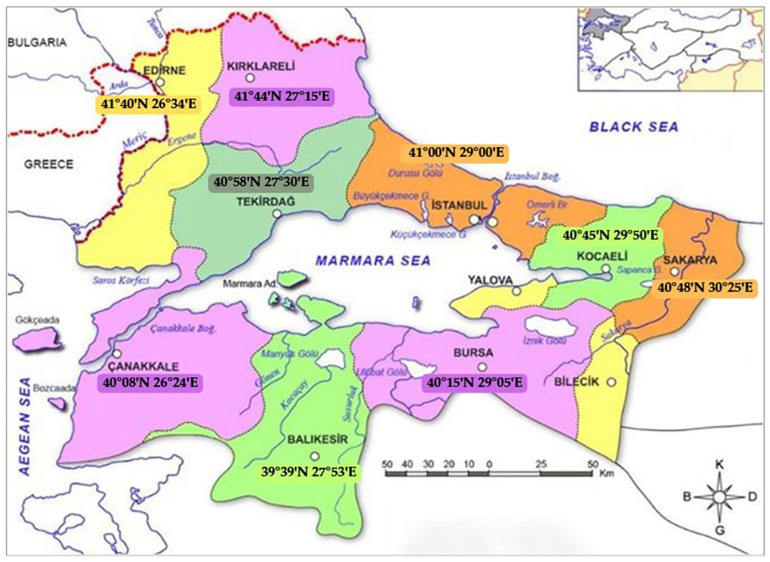
Map displaying the sampling regions (N = lat and E = long co-ordinates).

A total of 625 samples, consisting of 320 fishes, 200 mussels, 50 shrimps, 35 squids, 10 crabs, and 10 octopus, were collected from various provinces in the Marmara region between March 2020 and November 2022 (Figure 1 and Table 1). The samples were transported under cold chain conditions (4 °C ± 1 °C) to the laboratories of İstanbul University-Cerrahpaşa, Faculty of Veterinary Medicine, Department of Food Hygiene and Technology. Upon arrival, the samples were visually examined under sterile conditions and samples showing no signs of deterioration were immediately subjected to microbiological analysis. The remaining portions of each sample were transferred to separate Eppendorf tubes and stored at −20 °C for subsequent heavy metal analysis.

### 2.2. Heavy Metal Analysis

The concentrations of heavy metal residues, including lead (Pb), cadmium (Cd), mercury (Hg), total arsenic (As_tot_), and copper (Cu), in samples were analyzed using Inductively Coupled Plasma-Optical Emission Spectrometry (ICP-OES) with the Thermo iCAP 6000 series instrument (TS 3606/2008) (Thermo Fisher Scientific, Waltham, MA, USA). For inorganic arsenic (As_in_), this study assumed that it constitutes 10% of the total arsenic content, based on the findings of [34,35]. The ICP-OES method parameters used for the determination of heavy metal elements are summarized in Table 2.

Standard solutions (Chem-Lab NV, Zedelgem, Belgium) were prepared by combining standard stock solutions (1000 μg/dL) for each element to be analyzed. A 3-point calibration was conducted using standard and blank solutions as reference points. Following calibration, the samples were homogenized and transferred to graduated, heat-resistant glass tubes for analysis. Toxic elements were prepared for measurement using the wet combustion method. Hg was analyzed using a hydride generation system, while As, Cu, Pb, and Cd were analyzed without the hydride system.

Briefly, a 0.5 g sample was homogenized and placed in heat-resistant glass tubes. The wet combustion method involved adding 65% nitric acid (1 mL; HNO_3_; Merck, Darmstadt, Germany), mixing thoroughly, and allowing the reaction to proceed for 15–20 min. The mixture was heated at 100–120 °C for one hour, cooled, and then treated with 65% perchloric acid (1 mL; HClO_4_; Panreac, Barcelona, Spain) before undergoing a second heating cycle. After cooling, the samples were diluted with distilled water for the ICP-OES analysis of As, Cu, Pb, and Cd, while Hg was prepared using 10% hydrochloric acid (HCl; Merck, Darmstadt, Germany).

The results were expressed in mg/kg of sample, with triplicate measurements performed to ensure accuracy. The performance of the ICP-OES method was verified based on the parameters reported in Table 3.

### 2.3. Microbiological Analysis

The samples were analyzed for the detection of *Salmonella* spp. and *Listeria monocytogenes* according to ISO 6579-1:2017/AMD 1:2020 [36] and ISO 11290-1:2017 [37], respectively. The identification of *Salmonella* spp. was performed using the API 20E (bioMerieux, Nürtingen, Germany) biochemical test kit and *Salmonella* antisera (O and H-Vi polyvalent antisera, Denka, Niigata, Seiken). For *L. monocytogenes*, the Microbact™ TM 12L *Listeria* identification system (MB1128; Basingstoke, Oxoid) was applied. Subsequently, the isolates were subjected to molecular confirmation via PCR.

#### Molecular Confirmation by PCR

The genomic DNA isolates were extracted using the Roche High Pure PCR Template Preparation Kit (Roche, Mannheim, Germany) and stored at −20 °C until PCR analyses were conducted. The presence of the *inv*A gene, specific to *Salmonella* spp., was determined using the primers 5′-GTGAAATTATCGCCACGTTCGGGCAA-3′ and 5′-TCATCGCACCGTCAAAGGAACC-3′, following the method proposed by Rhan et al. [38]. *Salmonella enterica* subsp. *enterica serovar Typhimurium* (ATCC 14028) and *Salmonella enterica* subsp. *enterica serovar Enteritidis* (ATCC 13076) were used as the positive control, while *E. coli* (ATCC 25922) was used as the negative control. The confirmation of *L. monocytogenes* was carried out by targeting the specific internalin (*inl*A) gene for this pathogenic bacterium using the method proposed by Ingianni et al. [39] (inlA-fwd: 5′-ACTATCTAGTAACACGATTAGTGA-3′ and inlA-rev: 5′-CAAATTTGTTAAAATCCCAAGTGG-3′). *L. monocytogenes* (ATCC 7644) strain was used as the positive control, while *Staphylococcus aureus* (ATCC 29213) and *E. coli* (ATCC 25922) strains were used as negative controls in the study.

### 2.4. Statistical Analysis

Descriptive statistics were performed using SPSS 25.0 (SPSS Inc., Chicago, IL, USA) to present data as means and standard deviations (SD). Additionally, the prevalence of non-compliant samples and a 95% confidence interval (CI) were calculated. Data on HM levels in different fish samples were analyzed using one-way ANOVA followed by Tukey’s test. Comparisons between squids and octopus, as well as between shrimps and crabs, were performed using the Independent Samples *t*-test. In all statistical analyses, a *p*-value of < 0.05 was considered indicative of statistical significance. Furthermore, graphs were generated using GraphPad software Version 8.0 (GraphPad, San Diego, CA, USA).

The Provisional Tolerable Weekly Intake (PTWI), Estimated Daily Intake (EDI), and Estimated Weekly Intake (EWI) were assessed and computed. PTWI refers to the maximum allowable weekly consumption of trace and toxic metals from seafood, established by the Joint FAO/WHO Expert Committee on Food Additives [40] to prevent potential health risks. PTWI values indicate the safe threshold levels of metals in seafood products. To evaluate this, EDI and EWI were determined using Equations (1) and (2), respectively. If the EWI is below the PTWI, the consumption of seafood products is considered to pose minimal health risks [41].

(1) $$EDI\;\left(\frac{\mathrm{mg}}{\mathrm{kg}-\mathrm{day}}\right)=\frac{mMC\;\left(mg/kgww\right)\times Dfc\;}{BW\;(kg)}$$

(2) $$EWI\;\left(\frac{\mathrm{mg}}{\mathrm{kg}-\mathrm{week}}\right)=\frac{mMC\;\left(mg/kgww\right)\times Wfc\;}{BW\;(kg)}$$

*mMC* represents the mean concentration of metals, *Wfc* is the average daily (Equation (1)) or weekly (Equation (2)) seafood consumption per person in Türkiye [42], and *BW* is the average adult and children body weight, assumed to be 70 and 30 kg, respectively.

Moreover, the Target Hazard Quotient (THQ) and Hazard Index (HI) were calculated.

THQ is a key metric in health risk assessments, utilized to measure the potential intake of non-carcinogenic contaminants. It is computed using Equation (3), while the contaminant intake is determined through Equation (4). HI represents the cumulative sum of THQs for multiple chemicals under investigation [41]. A THQ value below 1 implies no significant risk to human health, whereas a value exceeding 1 indicates the possibility of adverse health effects.

(3) $$THQ=\frac{Intake}{Rfd}$$

(4) $$Intake\;\left(mg{\;kg}^{-1}{\mathrm{day}}^{-1}\right)=\frac{mMC\times IR\times EF\times ED}{BW\times AT}$$

where *mMC* is the mean concentration of metals (mg/kg wet weight); *IR* denotes the ingestion rate, assumed to be 0.0278 kg of wet weight per day for the Turkish population [42]; *EF* stands for the exposure frequency, set at 365 days per year; *ED* refers to the exposure duration, assumed to be 65 years [41]; *BW* is the body weight, fixed at 70 kg for adult and 30 kg for children; *AT* is the averaging time, representing the period over which exposure is averaged, calculated as 23,725 days (365 days/year × *ED*); and *RfD* is the oral reference dose for heavy metal intake (mg kg^−1^ day^−1^).

The *RfD* values for metals are: As (0.0003), Cd (0.001), Cu (0.04), Hg (0.0005), Pb (0.004), as reported by [41,43,44].

Finally, the results obtained from the microbiological analyses were analyzed using the Risk Ranger tool (https://foodsafetyportal.eu/riskranger/rr_riskranger.html(accessed on 1 February 2025)) to estimate the risk posed by each pathogen in fresh and ready-to-eat (RTE) fishery products [45,46].

The Risk Ranger tool integrates responses from 11 key questions to generate three distinct risk assessments: (i) a risk score ranging from 0 to 100, (ii) an estimate of the annual number of illness cases within the specified population, and (iii) the daily probability of infection for individuals in the target group.

## 3. Results

### 3.1. Heavy Metal Results

**Figure 2:**
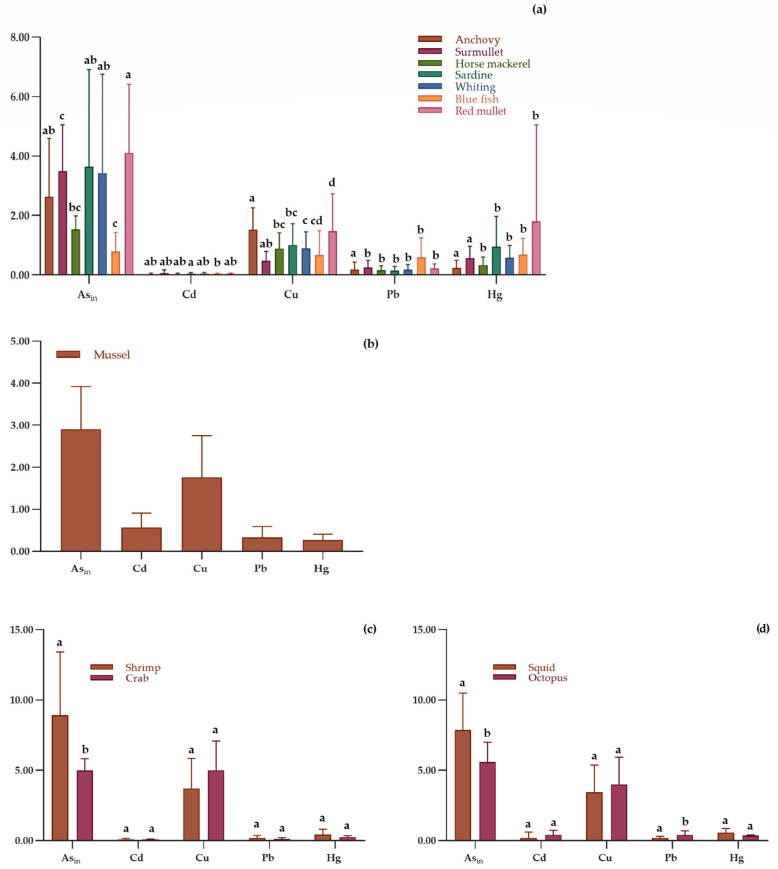
Means and ± SD of concentrations (mg/kg) of heavy metals in analyzed (**a**) fish species, (**b**) bivalve mollusks, (**c**) crustaceans, and (**d**) cephalopods. Bars with different letters in each group show significant difference at *p* < 0.05.

Figure 2 shows the concentrations (means ± SD; mg/kg) of heavy metals in fishery products and mussels. In general, regarding the fish samples within each group (group = HM), significant differences were consistently observed among the different sample types (*p* < 0.05). Regarding crustaceans, shrimps exhibited higher Asin levels than crabs (*p* < 0.05). A similar trend was observed for squids, which had a significantly higher Asin content than octopus but a lower Pb content (*p* < 0.05). However, comparisons for mussels could not be performed as they were the only samples within the bivalve mollusks category. All analyzed samples exceeded the permissible limits for Hg, Pb, and Cd (Table 4 and Table 5). Except for crabs, which remained below the regulatory thresholds for all three heavy metals, the results of anchovy, sardines, bluefish, shrimps, and octopus, were compliant with the EU limits for Cd.

All analyzed samples exceeded the PTWI values established for both adult and children (Tables S1–S4). Specifically, among fish samples, the EWI for adult (mg/week/70 kg) exceeded PTWI values, primarily for Hg. In mussels, Hg and Cd levels where high, whereas squids and octopus exhibited elevated concentrations of As_in_, Cd, and Hg. In shrimps, As_in_ and Hg were the predominant contaminants.

For children (mg/week/30 kg BW), the EWI values were particularly high in anchovy, horse mackerel, sardines, and whiting, with elevated levels of As_in_, Hg, and Pb. In crabs, As_in_, Hg, and Pb exceeded PTWI thresholds, while surmullet showed high levels of Hg and Pb. Bluefish and red mullet contained elevated concentrations of As_in_ and Hg.

Additionally, mussels exhibited high levels of As_in_, Cd, and Hg, while shrimps, squids, and octopus showed significant concentrations of As_in_, Cd, Hg, and Pb.

The Target Hazard Quotient (THQ) and Hazard Index (HI) values for both children and adult are presented in Tables S5–S8. In all samples, the THQ for adult remained below 1. However, in children, the THQ exceeded 1 for shrimps (THQAs_in_ = 1.948), crabs (THQAs_in_ = 1.094), squids (THQAs_in_ = 1.729), and octopus (THQAs_in_ = 1.226). As a result, the HI (sum of all THQs) in children exceeded 1 for these samples, as well as for mussels. In contrast, in adult, HI > 1 was observed only for shrimps.

### 3.2. Microbiological Results

The microbiological analysis revealed that surmullet, red mullet, crabs, mussels, and octopus tested negative for both *Salmonella* spp. and *Listeria monocytogenes*, while anchovy was negative only for *Salmonella* spp. (Table 6). Relating to bluefish, although suspicious colonies were detected in 20.00% of the samples during cultural isolation, PCR identification confirmed a negative result. All other samples, with varying detection percentages, were confirmed positive for both *Salmonella* spp. and *Listeria monocytogenes*. The risk ranking assessment indicated a risk score of 64 and 66 for *Salmonella* spp., and 73 and 71 for *Listeria monocytogenes*, respectively, in fresh fishery products and RTE fishery products (Table 7).

## 4. Discussion

### 4.1. Heavy Metals Contents in Fishery Products and Mussels

Heavy metals are environmental pollutants that cause serious damage to the ecological environment and accumulate in marine species such as mussels, fish, crustaceans and cephalopods. These accumulations in living organisms are a serious danger to human health [47].

In the present study, the prevalence of Hg, Pb, and Cd in mussel samples exceeded the MLs provided by Regulation (EU) No. 915/2023 by 5.00%, 0.50%, and 12.50%, respectively. The Sea of Marmara is an inland sea connected to both the Aegean Sea and the Black Sea and is highly exposed to pollution due to being the richest region in terms of agricultural and industrial areas and population density [48]. Indeed, consistent with Bat and Arici [48], our study revealed higher levels of Cd and Pb in mussels compared to fishery products from this area. This finding is likely due to both the high concentration of HMs in the environment and the filter-feeding nature of bivalves compared to fishery products. This result was also highlighted by Ozden et al. [49], who conducted a study in the Marmara region. Similarly, Güner et al. [50] found that Pb levels in mussels collected in Istanbul exceeded the regulatory limit, leading the authors to conclude that this could pose a public health risk.

In another study conducted by Koçbaş [51] on the northern coast of the Aegean Sea, the minimum and maximum values of heavy metals in black mussel *(M. galloprovincialis*) samples were reported as 0.53–7.96 mg/kg for Pb and 0.86–1.22 mg/kg for Cd [51]. In contrast, our mussel samples showed significantly lower Pb levels for both the minimum and maximum values (0.003–1.789 mg/kg), while for Cd (0.056–1.473 mg/kg) we observed lower minimum values but a higher maximum value. This suggests that Cd residues in this environment have remained relatively stable over time, while Pb residues have decreased.

Concerning As, Ocak [52] reported that total As levels in mussels from the Sea of Marmara ranged between 1.321 and 6.731 mg/kg, with inorganic As levels between 0.019 and 0.241 mg/kg. In our study, the concentration of As_in_ in mussels ranged from 0.685 to 7.827 mg/kg. Currently, no ML has been established for As_in_ in fishery products and bivalve mollusks, but the values found in this study were higher than those reported by Ocak [52]. Exposure induces various health hazards, with major target organs including the kidneys, lungs, liver, and skin. Severe As toxicity can lead to coma and death [53].

Mol and Alakavuk [54] reported that Hg was not detected in mussel samples from the Sea of Marmara, whereas the highest concentrations of heavy metals were Cu (3.473 mg/kg) and Cd (0.740 mg/kg). In the present study, Hg was detected in our samples (0.269 mg/kg), while the highest concentrations were found for Cu (5.122 mg/kg) and As (7.827 mg/kg), rather than Cd. On the other hand, Storelli et al. [55] determined Pb, Cd, and Hg concentrations in mussels (*M. galloprovincialis*) from the Ionian Sea in Italy as 1.19, 0.64, and 0.15 mg/kg, respectively, which were higher than the mean values found in our study, except for Hg. Topal and Önaç [47] emphasized that the increasing level of total As in Türkiye over the past decade is alarming and highlighted the need to establish a regulatory limit for this heavy metal.

With reference to fishery products, the Cd concentration in fish samples analyzed in this study ranged from 0.003 to 0.429 mg/kg, which is significantly lower than the levels (0.17–5.40 mg/kg) reported by Topal and Önaç [47]. Ramon et al. [56] reported that 99.4% of seafood (fish, crustaceans, and cephalopods) from the southeastern Mediterranean Sea fell within acceptable limits, with significantly higher total As concentrations in crustaceans and cephalopods than in fish. This finding aligns with our study’s results for total As. Additionally, high Hg levels were detected in *Mullus barbatus*, and similarly, in our study, Hg levels in red mullet were found to be relatively high (0.27 ± 0.17 mg/kg). Ramon et al. [56] also stated that increased mercury content in environmental resources is associated with industrial development, while the higher Hg levels in this species are linked to its ability to accumulate mercury due to its fatty and benthic nature. Monier et al. [57] indicated that heavy metal accumulation in fish organs (gray mullet, red sea bream and sardine) collected from the northern Mediterranean coast of Egypt followed the order Pb > Cu > Cd. In our study, heavy metal levels in red mullet samples followed the order Hg > Pb > Cd, whereas in sardines, the order was Hg > Pb, and Cd was not detected. Han et al. [58] reported mean concentrations of As, Cd, Hg, and Pb in marine fish from Zhejiang Province, China, as 0.783, 0.009, 0.031, and 0.043 mg/kg, respectively, indicating a potential health risk due to high As and Hg exposure. These results align with the Hg levels observed in fish samples from our study, leading us to conclude that continuous monitoring and consumer awareness initiatives are necessary.

### 4.2. Toxicological Repercussions

#### 4.2.1. Provisional Tolerable Weekly Intake (PTWI) and Estimated Daily and Weekly Intake (EDI/EWI) for Adult and Children

EDI and EWI values, as shown in Tables S1–S4 were assessed for adult (mg/week/70 kg BW) and children (mg/week/30 kg BW) and compared with the PTWI established by the Joint FAO/WHO Expert Committee on Food Additives [40]. The PTWI values for Hg and Cd were exceeded in the analyzed mussel samples, with a weekly Cd exposure of 1.12 mg for an adult (70 kg), well above the PTWI of 7 μg/kg body weight established by EFSA [59], suggesting a potential health risk associated with the consumption of these seafood products. Dogruyol et al. [2] reported an opposite situation, as their study in 2024 found that the EDI and EWI values for Cd and Hg in mussels collected from the Black Sea, Türkiye, were below permissible intake limits, representing less than 1% and 2% of the PTWI, respectively, indicating a negligible health risk. Ustaoğlu and Yüksel [60] ranked toxic elements as Cu > Pb > As > Cd based on EDI values and found that all EDI values were below the tolerable daily intake limit, thereby supporting the safety of consuming the analyzed fish species. Additionally, they reported Cd as having the lowest estimated daily intake value at 1.39 × 10^−3^ mg/kg BW/day. This finding is consistent with our study, in which the lowest estimated daily and weekly intake values for the examined fish species were also calculated for Cd. Abd-Elghany et al. [61] reported that the EDI of mercury in crab samples exceeded the tolerable daily intake limit by 152.17%, while the EDI values of Pb and Hg in shrimp samples surpassed the intake limits by 105.05% and 101.86%, respectively. Consequently, the potential non-carcinogenic risks associated with the ingestion of Hg and Pb through the consumption of these crab and shrimp species were highlighted. In the crab samples analyzed in the present study, the PTWI thresholds for children were exceeded for As_in_ (2.30 mg/week per 30 kg BW), Hg (0.55 mg/week per 30 kg BW), and Pb (1.15 mg/week per 30 kg BW). In shrimps, PTWI values for adult were exceeded for arsenic (1.75 mg/week per 70 kg BW) and Hg (0.5 mg/week per 70 kg BW). This non-compliant situation has significant public health implications, considering that the analysis was conducted by considering both adult and child exposure levels.

#### 4.2.2. Total Hazard Quotient (THQ) and Hazard Index (HI)

THQ is a measure used to evaluate the potential non-carcinogenic health risks associated with exposure to heavy metals (HMs). A THQ > 1 suggests that contaminant intake may pose a health concern, whereas a THQ < 1 indicates that intake is generally within safe limits. In this study, the THQ values calculated for each heavy metal in the analyzed fish species, mussels, crustaceans, and cephalopods were significantly below 1, except for As_in_, which exceeded 1 in squids and shrimps for children (Tables S5–S8). This finding suggests that As_in_, assumed to constitute 10% of total arsenic in this study, was the primary contributor to the overall hazard index (HI) for all metals (Tables S5–S8). Moreover, the HI value for all fish species was found to be below 1, whereas the HI values for shrimps in adult and for mussels, crabs, squids, and octopus in children were above 1. A HI value above 1 indicates that the cumulative non-carcinogenic risk from HMs in these seafood products exceeds safe limits, particularly for children.

Pertaining to the fish, Ustaoğlu and Yüksel [60] reported that THQ and HI values of all examined fish species (*Carassius gibelio*, *Squalis cephalus*, *Esoxlucius*, *and Tinca tinca*) were below 1, which is consistent with our findings. Dogruyol et al. [2] found that THQ values for all heavy metals examined in mussels (*M. galloprovincialis*) grown in three different regions of Türkiye were well below 1 and stated that they did not estimate a potential health risk that could arise from the consumption of farmed mussels in Türkiye. Similarly, in our study, THQ values of mussel samples were well below 1 for adult and children. In another study conducted in Türkiye, Ozden et al. [49] stated that the THQ values for the seafood they examined were within acceptable limits for Türkiye and EU countries. The highest THQ value was associated with the consumption of sea bream for Spanish individuals, while the lowest risk was determined for German consumers of seabass. The THQ value was not calculated above 1 for any of the fish species analyzed in the current study, while the highest risk was determined in red mullet. Likewise, Almafrachi et al. [62] indicated that none of the examined heavy metals (As, Cd, Cr, Fe, Mn, Ni, Se, Al and Zn) in 10 different fish species from Türkiye had THQ and HI values above 1. Therefore, they pointed out that this metal intake does not pose a health risk. Furthermore, Yu et al. [63] determined the highest THQ value for Hg (THQHg = 0.458), which was not at a level to pose a potential health risk. In our study, the highest THQ value for Hg was only valid for anchovy, surmullet, and sardine, while the highest THQ values in the analyzed seafood were generally determined for As. In addition, the HI value calculated to determine the accumulative effect of all heavy metals was below 1 [63], whereas the HI values of shrimps for adult and mussels, crabs, squids, and octopus for children were found to be above 1 in the present study. For Turkish individuals, the highest THQ value was associated with mussel consumption, while the low risk level in mussel consumption was associated with low consumption rates. In this context, the authors recommended that weekly consumption should not exceed 400 g, especially for individuals weighing < 70 kg, in order to prevent the health risk associated with mussel consumption.

### 4.3. Microbiological Semi-Quantitative Risk Assessment

*Salmonella* spp. and *L. monocytogenes* are the most frequent pathogens detected in seafood [21,64]. In the present study, the presence of *L. monocytogenes* and *Salmonella* spp. in fishery products and bivalves was also investigated and *Salmonella* spp. was found in fish at 4.69%, shrimps at 2.00%, squids at 2.90% and *L. monocytogenes* was found in fish at 6.25%, shrimps at 2.00%, and squids at 2.90%. As reported by Şeker et al. [25], the presence of *Salmonella* spp. was detected in 3% out of 627 mussel samples. However, in our study, all mussel samples tested negative for *Salmonella* spp. Phan et al. [65] declared that *Salmonella* spp. contamination was found in 24.5% of shrimp samples collected in Vietnam, while Morka and Hilda Abiola [66] stated that the incidence of *Salmonella* spp. in shrimps collected in Nigeria was almost half of this rate (11.5%) and Zarei et al. [67] reported a much lower rate of 4.3%. Meanwhile, in our study, *Salmonella* contamination in shrimp samples was determined to be 2.00%, which is much lower than the mentioned studies. Budiati et al. [68] found the presence of *Salmonella* spp. in raw fish samples to be 43.8%. However, *Salmonella* spp. was found to be 3.5% in 2.328 retail freshwater fish collected in China [69], which is close to the prevalence of *Salmonella* spp. (4.69%) in the fish samples analyzed in this study. Salama and Chennaoui [70] reported the prevalence of *Salmonella* spp. in seafood as 3% in mollusks in Spain, 10% in mussels and 9% in other seafood in Morocco, 7.4% in mollusks in Mexico, 1.5% in oysters in the USA, 18% in shellfish in Vietnam, 30.5% in fish, 29% in shrimps and 34.1% in oysters in India, and 1.6% in shrimps harvested in tropical marine waters of other Asian countries. Accordingly, the prevalence of *Salmonella* contamination in fishery products varies—from 0.3% in Italy to 36.6% in Vietnam—among different counties and originates from geographic locations, product species and types (raw or cooked), sources (imported vs. domestic), sampling stages (farm or retail stores), and parts (skin vs. intestine) [64].

In general, in our study, the incidence of *L. monocytogenes* was 0.00% in mussels and 4.24% in other fishery products, differing from the findings of Bouymajane et al. [71], who reported an incidence of 1.9% in raw fish in Meknes, Morocco. Additionally, Jegadeeshkumar et al. [72] reported that the presence of *L. monocytogenes* in seafood samples was 17.2%. Likewise, Peratikos et al. [21] found that 19 out of 165 (11.5%) fish samples in northern Greece were contaminated with *L. monocytogenes*. It was observed that these results, obtained by the researchers, were quite high when compared with the findings of the present study. Additionally, Dumen et al. [73] emphasized that 60 out of 700 (8.57%) samples collected in Istanbul were contaminated with *L. monocytogenes*, with the number of positive samples for raw fish, raw mussels, raw shrimps, and raw squids being 7.25%, 16%, 11%, and 4%, respectively. While the prevalence of *L. monocytogenes* in these fishes and squids was similar to our study, the prevalence in shrimps was much higher than our study findings. In a study conducted by Lufo et al. [74], the average prevalence of *L. monocytogenes* in shrimps obtained from Albania was 1.2%, which is slightly lower than our rate. On the other hand, Parlapani et al. [75] revealed that the incidence of *L. monocytogenes* was found to be 4.5% in fresh raw crab.

Risk analysis is the main step in minimizing the risk of public health hazards to an acceptable level and consists of three components including risk assessment, risk management, and risk communication. Risk assessment includes the evaluation of potential health effects that may be caused by exposure to foodborne hazards [76]. In this study, Risk Ranger, one of the risk ranking tools, was used to perform a microbiological semi-quantitative risk assessment. Using this tool, the response to eleven questions revealed the probability of illness per day per consumer of interest, the total predicted illnesses/annum in population of interest, and the risk ranking for both pathogens in seafood. The risk ranking is a value between 0 and 100, where risk ranking = 0 is equal to 1 case/10 billion people or less in 100 years, and risk ranking = 100 is the risk if food containing a lethal dose of the pathogen is consumed every day by all members of the population [46]. The risk benchmarks for Risk Ranger are categorized as low (risk score less than 32), moderate (risk score between 32 and 48), and high (risk score above 48) risk [77].

Risk analysis revealed that the risk associated with *Salmonella* spp. was slightly higher in ready-to-eat (RTE) fishery products (66 vs. 64). This result is due to the presence of post-processing control systems in fresh fishery products, where refrigeration alone acts as a control measure for *Salmonella* spp. Since *Salmonella* is a mesophilic microorganism, its growth is slowed at refrigeration temperatures. Additionally, fresh fishery products often undergo preparation before consumption (e.g., cooking), which typically eliminates pathogenic microorganisms, provided that proper cooking time and temperature are followed. These practices fall under good hygiene and food handling practices, whether at home or in food service establishments. Conversely, RTE products generally do not require further preparation before consumption, as they are pre-cooked. If the processing has been correctly performed by the producer, potential risks should be minimized. However, risk can never be entirely eliminated, and cross-contamination (e.g., contact with contaminated surfaces) can still occur, even during serving, potentially leading to recontamination issues. Regarding *Listeria monocytogenes*, the results were the opposite of those observed for *Salmonella* spp., with a slightly higher risk in fresh fishery products compared to RTE products (73 vs. 71). The main difference lies in the effectiveness of post-processing control systems. Unlike *Salmonella* spp., refrigeration does not act as a control measure for *L. monocytogenes*, as it is a psychrotrophic microorganism capable of growing at low temperatures. Other studies, although considering different parameters and matrices that are not directly comparable, have estimated risk scores for specific scenarios. For instance, the lowest risk score of zero was associated with the consumption of semi-preserved and frozen fish when histamine risk control measures were in place. In contrast, the highest risk score of 35 was linked to the consumption of fresh sardines in the absence of such control measures [77].

Bevilacqua et al. [76] assessed the risk ranking for *Listeria monocytogenes* in lettuce, consumed twice a week, and determined a score of 59, indicating a risk level that necessitates control measures. The study highlighted that risk ranking outcomes principally depend on factors such as consumption frequency, the characteristics of the consuming population, processing methods, and the extent of post-processing contamination. Additionally, the researchers reported that *E. coli* in chicken salad had a risk ranking of 40, representing a lower risk level that still requires preventive or control measures.

## 5. Conclusions

The metal concentrations identified in this study are of particular concern, as the PTWIs for arsenic As_in_, Hg, and Pb exceed recommended limits for children (mg/week/30 kg body weight) based on an estimated weekly intake. This is especially relevant for surmullet, bluefish, and red mullet—species frequently recommended for specific age groups and commonly consumed by children. Another critical finding for children is THQ greater than 1 for As_in_ in shrimps, crabs, squids, and octopus, as well as a THQ > 1 for HI. A THQ exceeding 1 indicates a potential risk of adverse health effects; however, it does not directly equate to a certainty of harm. Additionally, it is important to recognize that chemical exposure often involves mixtures of substances, whereas risk assessments typically focus on individual compounds. Moreover, the prevalence of the pathogens identified in this study underscores the need for rigorous monitoring, as they may pose a considerable health risk, particularly to consumers of fishery products and bivalve mollusks. Implementing monitoring and control programs is crucial to assess both heavy metal contamination and the presence of pathogenic microorganisms. Furthermore, raising awareness among food business operators and consumers regarding proper hygiene practices during handling and processing is essential for ensuring food safety.

## Figures and Tables

**Table 1:** Number of collected fishery products and bivalve mollusks.

Sample	Species Name	No. of Samples	Range of Sizes (cm)
Anchovy	*Engraulis encrasicolus*	64	11–18
Surmullet	*Mullus surmuletus*	39	15–25
Horse mackerel	*Trachurus trachurus*	82	11–18
Sardine	*Sardina pilchardus*	64	10–25
Whiting	*Merlangius euxmus*	40	20–40
Bluefish	*Pomatomus saltatrix*	15	15–18
Red mullet	*Mullus barbatus*	16	13 (17–20)
Mussel	*Mytilus galloprovincialis*	200	NA ^1^
Shrimp	*Parapenaeus longirostris*	50	NA ^1^
Crab	*Cancer pagurus*	10	NA ^1^
Squid	*Loligo vulgaris*	35	NA ^1^
Octopus	*Octopus vulgaris*	10	NA ^1^

^1^ NA—Data not available.

**Table 2:** ICP-OES method parameters for determination of heavy metal elements.

Parameters	Assigned Value
**Plasma gas flow rate**	15 L/min
**Argon carrier flow rate**	0.5 L/min
**Sample flow rate**	1.51 L/min
**The speed of peristaltic pump**	100 rpm
**RF Power**	1150 W

**Table 3:** The results of ICP-OES method validation for elements.

Elements	Quality Control (QC)	LoQ ^1^ (mg/kg)	Wavelength (nm)	Expected Concentration (ppm)	Measured Concentration (n = 3) (ppm)	Precision (RSD ^2^ %)	Recovery (%)
As	QC-1 QC-2 QC-3	0.023	189.042	0.500 1.000 5.000	0.505 0.998 5.014	0.946 0.125 0.381	101 99.8 100.28
Cd	QC-1 QC-2 QC-3	0.033	228.802	0.500 1.000 5.000	0.509 0.990 5.013	0.321 0.675 0.371	101.8 99 100.26
Pb	QC-1 QC-2 QC-3	0.0013	220.353	0.500 1.000 5.000	0.504 0.999 5.007	0.830 0.124 0.193	100.8 99.9 100.14
Cu	QC-1 QC-2 QC-3	0.028	324.754	0.500 1.000 5.000	0.502 1.001 4.994	0.575 0.983 0.174	100.4 100.1 99.88
Hg	QC-1 QC-2 QC-3	0.047	189.950	0.500 1.000 5.000	0.508 0.992 5.004	0.913 0.832 0.099	101.6 99.2 100.08

^1^ LoQ—limit of quantitation; ^2^ RSD—relative standard deviation.

**Table 4:** Maximum levels (MLs) for Hg, Pb, Cd, As, and Cu in fishery products and bivalves according to Regulation (EU) No. 915/2023 (consolidated text).

	MLs (mg/kg)				
	Hg	Pb	Cd	As_in_ ^b^	Cu ^b^
Fish (muscle meat)	0.50	0.30	0.05	-	-
Anchovy (*Engraulis* species) ^a^	0.30	0.30	0.25	-	-
Horse mackerel *(Trachurus trachurus)* ^a^	0.50	0.30	0.05	-	-
Surmullet (*Mullus surmuletus*) ^a^	1.00	0.30	0.05	-	-
Sardine (*Sardina pilchardus*)^a^	0.30	0.30	0.25	-	-
Whiting (*Merlangius merlangus*) ^a^	0.30	0.30	0.05	-	-
Red mullet (*Mullus barbatus*) ^a^	1.00	0.30	0.05	-	-
Bivalve molluscs	0.50	1.50	1.00	-	-
Crustaceans	0.50	0.50	0.50	-	-
Cephalopods	0.30	0.30	1.00	-	-

^a^ Fish species with explicitly defined limits. ^b^ No MLs are established in fishery products and bivalves for As_in_ and Cu.

**Table 5:** Prevalence of Hg, Pb, and Cd in fishery products and bivalves according to the MLs provided by Regulation (EU) No. 915/2023 (consolidated text).

Samples	No. Non-Compliant/Total No. of Samples (% Non-Compliant)		
	Hg (mg/kg)	Pb (mg/kg)	Cd (mg/kg)
Anchovy	17/64 (26.56)	8/64 (12.50)	-
CI_95_ (%) ^1^	17.30–38.48	6.47–22.77	
Surmullet	34/39 (87.18)	10/39 (25.64)	3/39 (7.69)
CI_95_ (%) ^1^	73.29–94.40	14.57–41.08	2.65–20.32
Horse mackerel	17/82 (20.73)	8/82 (9.76)	78/82 (95,12)
CI_95_ (%) ^1^	13.37–30.72	5.03–18.09	88.12–98.09
Sardine	55/64 (85.94)	9/64 (14.06)	-
CI_95_ (%) ^1^	75.38–92.42	7.58–24.62	
Whiting	32/40 (80.00)	4/40 (10.00)	10/40 (25.00)
CI_95_ (%) ^1^	65.24–89.50	3.96–23.05	14.19–40.19
Blue fish	15/15 (100.00)	5/15 (33.33)	-
CI_95_ (%) ^1^	79.61–100	15.18–58.29	
Red mullet	3/16 (18.75)	1/16 (6.25)	4/16 (25.00)
CI_95_ (%) ^1^	6.59–43.01	1.11–28.33	10.18–49.50
Mussel	10/200 (5.00)	1/200 (0.50)	25/200 (12.50)
CI_95_ (%) ^1^	2.74–8.96	0.09–2.78	8.61–17.80
Shrimp	9/50 (18.00)	5/50 (10.00)	-
CI_95_ (%) ^1^	9.77–30.80	4.35–21.36	
Crab	-	-	-
CI_95_ (%) ^1^			
Squid	31/35 (88.57)	4/35 (11.43)	1/35 (2.86)
CI_95_ (%) ^1^	74.05–95.46	4.54–25.95	0.51–14.53
Octopus	8/10 (80.00)	6/10 (60.00)	-
CI_95_ (%) ^1^	49.02–94.33	31.27–83.18	

^1^ CI_95_—95% confidence interval.

**Table 6:** Number of positive fishery products and bivalves for *Salmonella* spp. and *L. monocytogenes*/total number of samples (% positive) before cultural isolation and after PCR identification.

Sample	*Salmonella* spp.		*L. monocytogenes*	
	Cultural Isolation	PCR Identification ^a^	Cultural Isolation	PCR Identification ^a^
Anchovy	-	-	14/64 (21.88)	6/64 (9.38)
CI_95_ (%) ^1^			13.50–33.43	4.37–18.98
Surmullet	-	-	-	-
CI_95_ (%) ^1^				
Horse mackerel	13/82 (15.85)	8/82 (9.76)	16/82 (19.51)	6/82 (7.32)
CI_95_ (%) ^1^	9.51–25.26	5.03–18.09	12.38–29.37	3.40–15.06
Sardine	10/64 (15.63)	2/64 (3.13)	7/64 (10.94)	3/64 (4.69)
CI_95_ (%) ^1^	6.71–20.43	0.86–10.70	5.40–20.90	1.61–12.90
Whiting	7/40 (17.50)	4/40 (10.00)	4/40 (10.00)	1/40 (2.50)
CI_95_ (%) ^1^	8.75–31.95	3.96–23.05	3.96–23.05	0.44–12.88
Blue fish	4/15 (26.67)	1/15 (6.67)	3/15 (20.00)	-
CI_95_ (%) ^1^	10.90–51.95	1.19–29.82	7.05–45.19	
Red mullet	-	-	-	-
CI_95_ (%) ^1^				
Mussel	-	-	-	-
CI_95_ (%) ^1^				
Shrimp	3/50 (6.00)	1/50 (2.00)	7/50 (14.00)	1/50 (2.00)
CI_95_ (%) ^1^	2.06–16.22	0.35–10.50	6.95–26.19	0.35–10.50
Crab	-	-	-	-
CI_95_ (%) ^1^				
Squid	1/35 (2.86)	1/35 (2.86)	2/35 (5.71)	1/35 (2.86)
CI_95_ (%) ^1^	0.51–14.53	0.51–14.53	1.58–18.61	0.51–14.53
Octopus	-	-	-	-
CI_95_ (%) ^1^				
**Total (fishery products)**		17/425 (4.00)		18/425 (4.24)
**CI_95_ (%) ^1^**		2.50–6.30		2.70–6.59
**Total (bivalves)**		0/200 (0.00)		0/200 (0.00)
**CI_95_ (%) ^1^**		0.00–1.88		0.00–188

^a^ PCR identification was performed only for colonies suspected based on the cultural method; ^1^ CI_95_—95% confidence interval.

**Table 7:** Risk estimates for fresh and RTE fishery products for *Salmonella* spp. and *L. monocytogenes*.

	*Salmonella* spp.		*L. monocytogenes*	
	Fresh Fishery Products	RTE Fishery Products	Fresh Fishery Products	RTE Fishery Products
**Probability of illness per day per consumer of interest**	6.41 × 10^−2^	1.28 × 10^−1^	2.14 × 10^−1^	1.28 × 10^−1^
**Total predicted illnesses/annum in population of interest**	1.48 × 10^7^	2.96 × 10^7^	4.93 × 10^7^	2.97 × 10^7^
**RISK RANKING**	64	66	73	71

## Data Availability

Data will be made available on request.

## Supplementary Materials

The following supporting information can be downloaded at: https://www.mdpi.com/article/10.3390/toxics13030153/s1, Table S1: Results of the estimated daily and weekly intake and their comparison with the PTWI for the analyzed fish species; Table S2: Results of the estimated daily and weekly intake and their comparison with the PTWI for the analyzed bivalve mollusks; Table S3: Results of the estimated daily and weekly intake and their comparison with the PTWI for the analyzed crustaceans; Table S4: Results of the estimated daily and weekly intake and their comparison with the PTWI for the analyzed Cephalopods; Table S5: Target Hazard Quotient and Hazard Index for analyzed fish species; Table S6: Target Hazard Quotient and Hazard Index for analyzed bivalve mollusks; Table S7: Target Hazard Quotient and Hazard Index for analyzed crustaceans, Table S8: Target Hazard Quotient and Hazard Index for analyzed cephalopods.
