# Supplementary material for: Determination of Heavy Metal Levels and Assessment of L. monocytogenes and Salmonella spp. Presence in Fishery Products and Mussels from the Marmara Region, Türkiye
Source: Toxics. 2025 Feb 23;13(3):153. doi: 10.3390/toxics13030153 (PMC11945509; doi:10.3390/toxics13030153)
Supplement: Supplementary file 1 [file toxics-13-00153-s001.zip › toxics-3493834-supplementary.pdf]

Table S1. Results of the estimated daily and weekly intake and their comparison with the PTWI for the analyzed fish species.

**Sample**	**Heavy metals**	**PTWI[a]**	**PTWI[a]**	**PTWI[a]**	**EDI[b]**	**EDI[b]**	**EWI[c]**	**EWI[c]**
			**Adult**	**Children**	**Adult**	**Children**	**Adult**	**Children**
	As_{in}	0.015	1.05	0.45	0.12	0.27	0.81	**1.88**
	Cd	0.007	0.49	0.21	0.01	0.03	0.08	0.18
Anchovy	Cu	3.50	245	105	0.41	0.97	2.90	6.76
	Hg	0.004	0.28	0.12	0.51	1.18	**3.55**	**8.27**
	Pb	0.025	1.75	0.75	0.06	0.14	0.43	**1.01**
	As_{in}	0.015	1.05	0.45	0.02	0.05	0.15	0.36
	Cd	0.007	0.49	0.21	0.01	0.03	0.08	0.18
Surmullet	Cu	3.50	245	105	0.19	0.44	1.32	3.08
	Hg	0.004	0.28	0.12	0.19	0.45	**1.36**	**3.17**
	Pb	0.025	1.75	0.75	0.17	0.39	1.16	**2.71**
	As_{in}	0.015	1.05	0.45	0.07	0.17	0.51	**1.20**
	Cd	0.007	0.49	0.21	0.01	0.02	0.06	0.14
Horse mackerel	Cu	3.50	245	105	0.43	1.00	2.99	6.99
	Hg	0.004	0.28	0.12	0.06	0.15	**0.45**	**1.06**
	Pb	0.025	1.75	0.75	0.05	0.11	0.33	**0.78**
	As_{in}	0.015	1.05	0.45	0.10	0.23	0.69	**1.60**
	Cd	0.007	0.49	0.21	0.02	0.04	0.12	0.28
Sardine	Cu	3.50	245	105	0.14	0.32	0.95	2.21
	Hg	0.004	0.28	0.12	0.16	0.37	**1.10**	**2.57**
	Pb	0.025	1.75	0.75	0.07	0.16	0.49	**1.15**
	As_{in}	0.015	1.05	0.45	0.10	0.22	0.67	**1.57**
	Cd	0.007	0.49	0.21	0.01	0.03	0.08	0.18
Whiting	Cu	3.50	245	105	0.25	0.58	1.75	4.09
	Hg	0.004	0.28	0.12	0.16	0.38	**1.14**	**2.67**
	Pb	0.025	1.75	0.75	0.05	0.12	0.35	**0.83**
	As_{in}	0.015	1.05	0.45	0.04	0.10	0.30	**0.70**
	Cd	0.007	0.49	0.21	0.01	0.02	0.06	0.14
Blue fish	Cu	3.50	245	105	0.25	0.58	1.73	4.05
	Hg	0.004	0.28	0.12	0.09	0.22	**0.65**	**1.52**
	Pb	0.025	1.75	0.75	0.05	0.11	0.32	0.74
	As_{in}	0.015	1.05	0.45	0.10	0.24	0.72	**1.67**
	Cd	0.007	0.49	0.21	0.01	0.03	0.08	0.18
Red mullet	Cu	3.50	245	105	0.28	0.66	1.97	4.60
	Hg	0.004	0.28	0.12	0.27	0.62	**1.87**	**4.37**
	Pb	0.025	1.75	0.75	0.04	0.10	0.30	0.69

[a] Provisional tolerable weekly intake (mg/week/kg BW, Adult: mg/week/70 kg BW, Children: mg/week/30 kg BW)

[b] Estimated daily intake (Adult: mg/day/70 kg BW, Children: mg/day/30 kg BW).

[c] Estimated weekly intake (Adult: mg/week/70 kg BW, Children: mg/week/30 kg BW).

Table S2. Results of the estimated daily and weekly intake and their comparison with the PTWI for the analyzed bivalve mollusks.

Sample	Heavy metals	PTWI[a]	PTWI[a]	PTWI[a]	EDI[b]	EDI[b]	EWI[c]	EWI[c]
			**Adult**	**Children**	**Adult**	**Children**	**Adult**	**Children**
Mussel	As_{in}	0.015	1.05	0.45	0.08	0.19	0.57	**1.33**
	Cd	0.007	0.49	0.21	0.16	0.37	**1.12**	**2.62**
	Cu	3.50	245	105	0.50	1.16	3.47	8.09
	Hg	0.004	0.28	0.12	0.08	0.18	**0.53**	**1.24**
	Pb	0.025	1.75	0.75	0.09	0.22	0.65	1.52

[a] Provisional tolerable weekly intake (mg/week/kg BW, Adult: mg/week/70 kg BW, Children: mg/week/30 kg BW)

[b] Estimated daily intake (Adult: mg/day/70 kg BW, Children: mg/day/30 kg BW).

[c] Estimated weekly intake (Adult: mg/week/70 kg BW, Children: mg/week/30 kg BW).

Table S3. Results of the estimated daily and weekly intake and their comparison with the PTWI for the analyzed crustaceans.

Sample	Heavy metals	PTWI[a]	PTWI[a]	PTWI[a]	EDI[b]	EDI[b]	EWI[c]	EWI[c]
			**Adult**	**Children**	**Adult**	**Children**	**Adult**	**Children**
Shrimp	As_{in}	0.015	1.05	0.45	0.25	0.58	**1.75**	**4.09**
	Cd	0.007	0.49	0.21	0.02	0.05	0.16	**0.37**
	Cu	3.50	245	105	1.04	2.42	7.27	16.96
	Hg	0.004	0.28	0.12	0.05	0.12	**0.35**	**0.83**
	Pb	0.025	1.75	0.75	0.12	0.28	0.83	**1.93**
Crab	As_{in}	0.015	1.05	0.45	0.14	0.33	0.99	**2.30**
	Cd	0.007	0.49	0.21	0.02	0.05	0.16	0.37
	Cu	3.50	245	105	1.41	3.28	9.85	22.98
	Hg	0.004	0.28	0.12	0.03	0.08	0.24	**0.55**
	Pb	0.025	1.75	0.75	0.07	0.16	0.49	**1.15**

[a] Provisional tolerable weekly intake (mg/week/kg BW, Adult: mg/week/70 kg BW, Children: mg/week/30 kg BW)

[b] Estimated daily intake (Adult: mg/day/70 kg BW, Children: mg/day/30 kg BW).

[c] Estimated weekly intake (Adult: mg/week/70 kg BW, Children: mg/week/30 kg BW).

Table S4. Results of the estimated daily and weekly intake and their comparison with the PTWI for the analyzed Cephalopods.

Sample	Heavy metals	PTWI[a]	PTWI[a]	PTWI[a]	EDI[b]	EDI[b]	EWI[c]	EWI[c]
			**Adult**	**Children**	**Adult**	**Children**	**Adult**	**Children**
Calamari	As_{in}	0.015	1.05	0.45	0.22	0.52	**1.56**	**3.63**
	Cd	0.007	0.49	0.21	0.05	0.12	**0.37**	**0.87**
	Cu	3.50	245	105	0.97	2.25	6.76	15.77
	Hg	0.004	0.28	0.12	0.05	0.12	**0.35**	**0.83**
	Pb	0.025	1.75	0.75	0.12	0.28	0.83	**1.93**
Octopus	As_{in}	0.015	1.05	0.45	0.16	0.37	**1.10**	**2.57**
	Cd	0.007	0.49	0.21	0.11	0.26	**0.79**	**1.84**
	Cu	3.50	245	105	1.12	2.62	7.86	18.34
	Hg	0.004	0.28	0.12	0.12	0.27	**0.81**	**1.88**
	Pb	0.025	1.75	0.75	0.1	0.24	0.71	**1.65**

[a] Provisional tolerable weekly intake (mg/week/kg BW, Adult: mg/week/70 kg BW, Children: mg/week/30 kg BW)

[b] Estimated daily intake (Adult: mg/day/70 kg BW, Children: mg/day/30 kg BW).

[c] Estimated weekly intake (Adult: mg/week/70 kg BW, Children: mg/week/30 kg BW).

Table S5. Target Hazard Quotient and Hazard Index for analyzed fish species.

Sample	Heavy metals	THQ[a] Adult	THQ[a] Children
Anchovy	As_{in}	0.029	0.067
	Cd	0.003	0.007
	Cu	0.103	0.241
	Hg	0.127	0.296
	Pb	0.015	0.036
**HI[b]**		0.28	0.65
Surmullet	As_{in}	0.005	0.013
	Cd	0.003	0.007
	Cu	0.047	0.110
	Hg	0.049	0.113
	Pb	0.042	0.097
**HI[b]**		0.15	0.34
Horse mackerel	As_{in}	0.018	0.043
	Cd	0.002	0.005
	Cu	0.107	0.250
	Hg	0.016	0.038
	Pb	0.012	0.028
**HI[b]**		0.16	0.36
Sardine	As_{in}	0.025	0.057
	Cd	0.004	0.010
	Cu	0.034	0.079
	Hg	0.039	0.092
	Pb	0.018	0.041
**HI[b]**		0.12	0.28
Whiting	As_{in}	0.024	0.056
	Cd	0.003	0.007
	Cu	0.063	0.146
	Hg	0.041	0.095
	Pb	0.013	0.030
**HI[b]**		0.14	0.33
Blue fish	As_{in}	0.011	0.025
	Cd	0.002	0.005
	Cu	0.062	0.144
	Hg	0.023	0.054
	Pb	0.011	0.026
**HI[b]**		0.11	0.25
Red mullet	As_{in}	0.026	0.060
	Cd	0.003	0.007
	Cu	0.070	0.164
	Hg	0.067	0.156
	Pb	0.011	0.025
**HI[b]**		0.80	0.41

[a]Target hazard quotient; [b]Hazard index.

Table S6. Target Hazard Quotient and Hazard Index for analyzed bivalve mollusks.

Sample	Heavy metals	THQ[a] Adult	THQ[a] Children
Mussels	As_{in}	0.272	0.635
	Cd	0.160	0.374
	Cu	0.012	0.029
	Hg	0.152	0.355
	Pb	0.023	0.054
**HI[b]**		0.62	1.45

[a]Target hazard quotient; [b]Hazard index.

Table S7. Target Hazard Quotient and Hazard Index for analyzed crustaceans.

Sample	Heavy metals	THQ[a] Adult	THQ[a] Children
Shrimps	As_{in}	0.835	1.948
	Cd	0.023	0.053
	Cu	0.026	0.061
	Hg	0.101	0.236
	Pb	0.030	0.069
**HI[b]**		1.01	2.37
Crab	As_{in}	0.469	1.094
	Cd	0.023	0.053
	Cu	0.035	0.082
	Hg	0.068	0.158
	Pb	0.018	0.041
**HI[b]**		0.61	1.43

[a]Target hazard quotient; [b]Hazard index.

Table S8. Target Hazard Quotient and Hazard Index for analyzed cephalopods.

Sample	Heavy metals	THQ[a] Adult	THQ[a] Children
Calamari	As_{in}	0.741	1.729
	Cd	0.053	0.125
	Cu	0.024	0.056
	Hg	0.101	0.236
	Pb	0.030	0.069
**HI[b]**		0.95	2.22
Octopus	As_{in}	0.525	1.226
	Cd	0.113	0.263
	Cu	0.028	0.066
	Hg	0.231	0.538
	Pb	0.025	0.059
**HI[b]**		0.92	2.15

[a]Target hazard quotient; [b]Hazard index.
